# Effects of gender, digit ratio, and menstrual cycle on intrinsic brain functional connectivity: A whole‐brain, voxel‐wise exploratory study using simultaneous local and global functional connectivity mapping

**DOI:** 10.1002/brb3.890

**Published:** 2017-12-19

**Authors:** Tomohiro Donishi, Masaki Terada, Yoshiki Kaneoke

**Affiliations:** ^1^ Department of System Neurophysiology Graduate School of Wakayama Medical University Wakayama Japan; ^2^ Wakayama‐Minami Radiology Clinic Wakayama Japan

**Keywords:** 2D:4D digit ratio, fMRI, menstrual cycle, normalized alpha centrality, prenatal sex hormones

## Abstract

**Introduction:**

Gender and sex hormones influence brain function, but their effects on functional network organization within the brain are not yet understood.

**Methods:**

We investigated the influence of gender, prenatal sex hormones (estimated by the 2D:4D digit ratio), and the menstrual cycle on the intrinsic functional network organization of the brain (as measured by 3T resting‐state functional MRI (rs‐fMRI)) using right‐handed, age‐matched university students (100 males and 100 females). The mean (±*SD*) age was 20.9 ± 1.5 (range: 18–24) years and 20.8 ± 1.3 (range: 18–24) years for males and females, respectively. Using two parameters derived from the normalized alpha centrality analysis (one for local and another for global connectivity strength), we created mean functional connectivity strength maps.

**Results:**

There was a significant difference between the male mean map and female mean map in the distributions of network properties in almost all cortical regions and the basal ganglia but not in the medial parietal, limbic, and temporal regions and the thalamus. A comparison between the mean map for the low 2D:4D digit ratio group (indicative of high exposure to testosterone during the prenatal period) and that for the high 2D:4D digit ratio group revealed a significant difference in the network properties of the medial parietal region for males and in the temporal region for females. The menstrual cycle affected network organization in the brain, which varied with the 2D:4D digit ratio. Most of these findings were reproduced with our other datasets created with different preprocessing steps.

**Conclusions:**

The results suggest that differences in gender, prenatal sex hormone exposure, and the menstrual cycle are useful for understanding the normal brain and investigating the mechanisms underlying the variable prevalence and symptoms of neurological and psychiatric diseases.

## 1 INTRODUCTION

Sex hormones influence brain development (Berenbaum & Beltz, 2011; McCarthy, Pickett, VanRyzin, & Kight, 2015; Savic, Frisen, Manzouri, Nordenstrom, & Linden Hirschberg, 2017; Van Hemmen, Saris et al., 2016), activity, and function (Chen, Decety, Huang, Chen, & Cheng, 2016; Crewther, Cook, Kilduff, & Manning, 2015; Goldstein, Jerram, Abbs, Whitfield‐Gabrieli, & Makris, 2010; Petersen, Kilpatrick, Goharzad, & Cahill, 2014; Sundstrom Poromaa & Gingnell, 2014; Thimm, Weis, Hausmann, & Sturm, 2014; Toffoletto, Lanzenberger, Gingnell, Sundstrom‐Poromaa, & Comasco, 2014; Van Hemmen, Veltman et al., 2016; Weis, Hausmann, Stoffers, & Sturm, 2011; Weis et al., 2008; Zhu, Kelly, Curry, Lal, & Joseph, 2015). Elucidating the mechanisms underlying this influences is vital because many neurological and psychiatric diseases exhibit gender‐dependent variability in both their prevalence and symptoms (Bao & Swaab, 2010; Cahill, 2006; McCarthy, Arnold, Ball, Blaustein, & De Vries, 2012; Zagni, Simoni, & Colombo, 2016). Moreover, menstrual cycle affects neurological and psychiatric symptoms in females, including the incidence of epileptic seizures and depressive states (Backstrom et al., 2003).

Although gender differences in brain structure (Ruigrok et al., 2014), structural connectivity (Ingalhalikar et al., 2014), and functional connectivity (Scheinost et al., 2015) have been previously reported, the extent of these differences (such as Cohen's d) is often too small to distinguish male and female distributions (Joel et al., 2015), and some results are inconsistent. One possibility is that the effects of gender on the functional connectivity of the brain are subtler than the effects of other factors, such as interindividual variability due to age, educational level, handedness, menstrual cycle, diurnal variance, and mental state during the measurement. Another possibility is that the functional network organization of the female brain is essentially the same as that of the male brain, but there is a shift outside of the normal distribution for females due to the variable effects of sex hormones throughout the menstrual cycle. Furthermore, rs‐fMRI data processing and parameters used in previous studies could be associated with different sensitivities.

In this study, we investigated the effects of gender on the functional network organization of the brain using data from age‐matched, right‐handed, university students. If there were any statistically significant differences between males and females, we then investigated the effects of prenatal sex hormones on the functional network organization for both the male and female groups. The magnitude of prenatal sex hormone exposure was estimated using the right‐hand 2D:4D digit ratio (Honekopp & Watson, 2010; Manning, Bundred, Newton, & Flanagan, 2003; Manning, Kilduff, Cook, Crewther, & Fink, 2014; Zheng & Cohn, 2011). We also investigated the effects of the menstrual cycle on the functional network organization of the female brain.

To this end, we used data‐driven, voxel‐wise, whole‐brain functional network analysis using normalized alpha centrality (nAC) (Ghosh & Lerman, 2011). This method is useful for detecting regional network properties with high spatial resolution without the a priori determination of nodes and networks to measure functional connectivity. Furthermore, both local and global connectivity (GC) strengths determined by this method can be directly compared in a meaningful way, which will have greater sensitivity to detect subtle differences in network organizations than single parameters, such as degree or eigenvector centrality (Buckner et al., 2009; Lohmann et al., 2010) and GC (Cole, Pathak, & Schneider, 2010; Ueyama et al., 2013; Yang et al., 2014).

In this study, we hypothesized that two groups categorized by gender, the 2D:4D digit ratio, or the menstrual cycle would constitute distinct populations, resulting in significant differences in mean brain organizations. First, we created the mean brain network property map for each group. At each gray‐matter voxel, we calculated the mean values of local and global network parameters and classified its node property (global hub, global node, local hub, or local node) based on these values. To compare the two mean images, we calculated the number of nodes with each node property in each brain region and investigated differences in its distribution. We identified 14 brain regions using automated anatomical labeling (AAL) (Tzourio‐Mazoyer et al., 2002). Significant differences between these two mean maps indicated that the functional networks of male brains constitute a population different from that of females. Similarly, we investigated the effects of prenatal sex hormones, and the menstrual cycle, by dividing the group into two subgroups by the 2D:4D digit ratios (low and high digit ratio groups) and by menstrual phases (follicular and luteal phase groups). Finally, we investigated the effects of rs‐fMRI data preprocessing on our brain network organization maps.

## 2 MATERIALS AND METHODS

### 2.1 Participants

This study was approved by the Ethics Committee of Wakayama Medical University, and all participants provided written informed consent. We recruited 200 right‐handed Japanese student volunteers who ranged in age from 18 to 24 years (100 males and 100 females) from the university in Wakayama City; the authors do not belong to this university. The participants were recruited by posters that described objective of this study and advertised the recruitment of participants. Thus, the participants independently decided to take part in the study and received 6,000 yen (approximately 50 US dollars) as a reward.

The Edinburgh Handedness Inventory score (Oldfield, 1971) was used to determine the handedness of our participants; all scores were ≥70. The mean age and other demographic data are shown in Table 1. The health status of each participant was checked using questionnaires. No participants were taking medications, including oral contraceptives, or had a history of severe head injuries. Female participants were asked to report the date of their last menstrual period in order to estimate their menstrual phase (follicular or luteal) at the time of MRI acquisition. We classified females as being in the follicular phase if they were between day 1 and 14 and in the luteal phase if they were between days 15 and 28. Data for seven participants who stated their last menstrual period occurred more than 28 days ago were not used for the analysis of menstrual phase effects because it was difficult to estimate the menstrual cycle date on the experiment day.

**Table 1:** Demographic data

	Male, *N* = 100	Female, *N* = 100	*p* value
Age [year]	20.92 ± 1.47	20.77 ± 1.29	.45*
Edinburgh Handedness score	91.17 ± 8.35	94.62 ± 7.12	.0011**
GM volume (ml)	806 ± 52	730 ± 46	1.04 × 10^−21^ *
GM ratios to TIV	0.518 ± 0.022	0.533 ± 0.021	1.64 × 10^−6^ *
Digit ratio	0.938 ± 0.026	0.951 ± 0.030	.0018*
Mean Power's FD	0.127 ± 0.043	0.127 ± 0.045	.992*
Menstrual cycle [day]		6.36 ± 5.34 (FOL, *N* = 53)21.38 ± 3.64 (LUT, *N* = 40)39.14 ± 10.01 (unknown, *N* = 7)	

*t test or **Mann–Whitney *U* test for gender differences. FOL, follicular phase; LUT, luteal phase; TIV, total intracranial volume.

### 2.2 Digit ratio measurement

To measure each participant's digit length, we scanned an image of the right hand using a portable color scanner (CanoScan Lide 210, Canon Inc., Tokyo, Japan) with a spatial resolution of 4,800 × 4,800 dpi. The second (2D) and fourth digit (4D) lengths were measured from the proximal finger crease to the distal tip of the finger using a digital Vernier caliper to the nearest 0.01 mm, as described in a previous study (Kaneoke, Donishi, Iwahara, & Shimokawa, 2017; Manning, Scutt, Wilson, & Lewis‐Jones, 1998). In this study, we investigated the right‐hand digit ratio based on the previous studies; these studies showed that prenatal sex hormones affect the right‐hand digit ratio more than the left‐hand digit ratio (Honekopp & Watson, 2010; Manning et al., 1998, 2014). To investigate the effect of the digit ratio on brain network properties, we divided our male and female groups into two subgroups (low and high digit ratio groups) based on the median values for each group. The number of participants and mean ages are shown in Table 2.

**Table 2:** Subgroups based on digit ratios

Digit ratio	Male, rangeage (*N*)	Female, rangeage (*N*)	Female menstrual phase		
			FOL	LUT	*p* value*
Low	0.865–0.93720.7 ± 1.6 (47)	0.858–0.95121.0 ± 1.4 (49)	0.858–0.95020.9 ± 1.3 (27)	0.875–0.94921.2 ± 1.5 (18)	.52
High	0.938–1.00721.1 ± 1.3 (53)	0.951–1.01620.5 ± 1.2 (51)	0.953–1.01620.7 ± 1.2 (26)	0.951–1.01620.4 ± 1.2 (22)	.47
*p* value**	.13	.057	.50	.078	

*t test for age between FOL and LUT. **t test for age between LOW and HIGH. FOL, follicular phase; LUT, luteal phase.

### 2.3 MRI data acquisition

All the participants underwent MRI acquisition on a weekday afternoon (mean ± *SD *= 4 p.m. ± 26 min, ranging from 1 p.m. to 5 p.m.); timing was important because of the diurnal fluctuation in the brain activity (Hodkinson et al., 2014). A 3 Tesla MRI (PHILIPS, the Netherlands) with a 32‐channel head coil (SENSE‐Head‐32CH) was used to acquire structural and resting‐state functional images of the brain. T1‐weighted structural images were obtained with the following parameters: TR = 6.9 ms, TE = 3.3 ms, FOV = 256 mm, matrix scan = 256, slice thickness = 1.0 mm, and flip angle = 10°. Functional images were collected using a gradient‐echo echo‐planar pulse sequence sensitive to BOLD contrast (Ogawa, Lee, Kay, & Tank, 1990) with the following parameters: TR = 3,000 ms, TE = 30 ms, FOV = 192 mm, matrix scan = 64, slice thickness = 3.0 mm, and flip angle = 80°. Three runs, which each comprised 107 volumes, were performed on each participant. In total, data were acquired for approximately 15 min during a resting state for each participant, as this duration was deemed the most appropriate to obtain reliable data (Birn et al., 2013). The participants were then asked to stay awake with their eyes closed during image acquisition.

Soon after acquisition, the participants were asked to describe what they were thinking about during image acquisition using the resting‐state questionnaire (ReSQ) developed by Tzourio‐Mazoyer et al. (Delamillieure et al., 2010). We then checked for gender differences in interoception data to investigate the effect of mental activity on gender differences in regional brain network property patterns.

### 2.4 MRI data preprocessing

**Figure 1:**
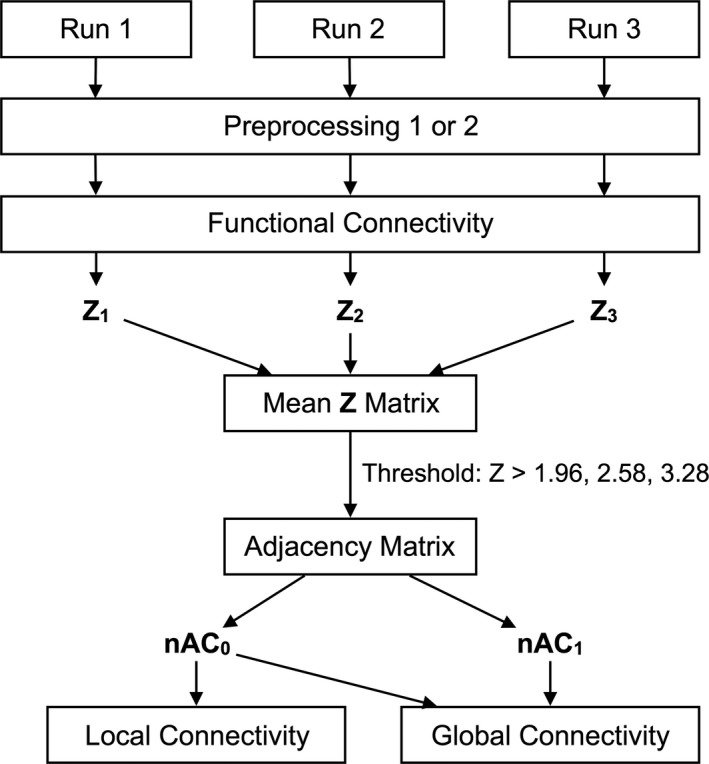
Work flow of the study. Three rs‐fMRI runs (107 consecutive image volumes) were acquired. For each session, we performed preprocessing, including slice‐timing correction, head motion correction, denoising, temporal filtering, image normalization, and gray‐matter segmentation. For preprocessing 1, denoising included CompCor and the regression of six head motion parameters and global signal. Denoising for preprocessing 2 included CompCor. Then, the functional connectivity strength of each pair of gray‐matter voxels was calculated and transformed to *Z* values for each run (*Z*1, *Z*2, and *Z*3). These 3 values were averaged (*Z* = (*Z*1 + *Z*2 + *Z*3)/3). An adjacent matrix was then determined using the *Z* matrix with three different thresholds (1.96, 2.58, or 3.28) to calculate normalized alpha centrality (nAC_0_ and nAC_1_) at each gray‐matter voxel. Global connectivity (GC) was the difference between the two values (nAC_1_–nAC_0_)

An outline of the work flow is shown in Figure 1. Functional data were preprocessed using SPM8 (http://www.fil.ion.ucl.ac.uk/spm) and in‐house software developed with MATLAB (MathWorks, Natick, MA, USA). The first five volumes of each fMRI acquisition run were discarded to allow for T1‐equilibration effects, thus leaving 102 consecutive volumes. Slice timing was adjusted to the topmost slice (acquired last): First, the number of time‐course data points (*n* = 102) for each voxel signal was increased to 68 (number of slices) using spline interpolation. Then, 101 data points at the same timing as the last slice for each image volume were chosen for each voxel. Rigid body translation and rotation were performed in SPM8 to correct for head motion, followed by spatial normalization by 12‐parameter affine transformation according to the International Consortium for Brain Mapping Echo‐Planar Imaging template. A session showing either a translation of ≥2 mm or a rotation of ≥0.02 radian was excluded from further analysis. A summary of the excluded sessions is shown in Table S1.

Images were then resampled to 2‐mm isotropic voxels and spatially smoothed using an 8 mm full width at half‐maximum Gaussian kernel. To exclude *nuisance* signals unrelated to brain activity, we used CompCor (Behzadi, Restom, Liau, & Liu, 2007), six head motion time‐course parameters regression (Power, Schlaggar, & Petersen, 2015), and global signal regression (the mean time course of the functional brain image voxels) (Power, Plitt, Laumann, & Martin, 2016). Preprocessing, including CompCor, head motion regression, and global signal regression, is called “preprocessing 1” in this study. Temporal filtering (bandpass ranging from 0.01 to 0.1 Hz) was applied to remove constant offset and linear trends over each run. Structural images were normalized and resampled using the same method as fMRI data preprocessing and then segmented into probability maps of gray matter using SPM8. The magnitude of head motion was calculated using Power's framewise displacement (FD) (Power et al., 2015), and gender differences were assessed.

We also created another set of the functional data that had undergone preprocessing without head motion and global signal regression, which we call “preprocessing 2” in this text. Table 3 shows the database sets used in this study. The results for these datasets are shown in Supporting Information.

**Table 3:** Database and mean network parameters used in this study

Preprocess	*Z* threshold	nAC_0_	nAC_1_	GC
1	2.58	0.0169 ± 0.0060	0.0169 ± 0.0086	0.0000 ± 0.0031
1	1.96	0.0169 ± 0.0046	0.0169 ± 0.0071	0.0000 ± 0.0030
1	3.28	0.0169 ± 0.0074	0.0169 ± 0.0118	0.0000 ± 0.0059
2	2.58	0.0169 ± 0.0052	0.0169 ± 0.0068	0.0000 ± 0.0021

Preprocessing 1 included CompCor, head motion regression, and global signal regression. Preprocessing 2 included CompCor. The *Z* threshold was used to create an adjacency matrix. The values of nAC_0_, nAC_1_, and GC are shown as the mean ± *SD* of all the participant data.

### 2.5 Selection of gray‐matter voxels in the functional images

The gray‐matter mask image (2‐mm cubic voxel) was created as follows. First, a gray‐matter structural image at zero threshold was used to create a binary gray‐matter image. All the voxels in the image outside of the regions defined by (AAL) (Tzourio‐Mazoyer et al., 2002) were excluded to remove nonbrain tissues such as the venous sinuses. Because the gray‐matter image discriminated by SPM8 lacks most of the basal ganglia (BG) and thalamus (TH) voxels, we added the voxels within these structures as defined by AAL. Second, for each participant, all gray‐matter binary images were averaged to produce a probability map, and the voxels with a probability < 90% were removed. This gray‐matter mask image was then used to extract gray‐matter voxels from the functional images for each participant. The extracted functional images were then down‐sampled to 6‐mm cubic voxels. Finally, voxels with nonzero signals for all participants were included, which resulted in 5,916 voxels of gray‐matter functional images for all participants. Each voxel was treated as a node of the brain functional network.

### 2.6 Gray‐matter volume measurement

We measured gray‐matter volume for each participant using T1‐weighted MRI to check the effect of the value on the network properties. Voxel‐based morphometry analysis in SPM was used to measure gray matter and total intracranial volume (TIV) according to a previous study (Chen, Sachdev, Wen, & Anstey, 2007).

### 2.7 Network property analysis

Functional connectivity between two gray‐matter voxels was calculated by Pearson's correlation coefficient (*r*) using the time‐course data for the two voxels in the functional images. The value of *r* was then converted to a *Z* value after the effective sample size correction using the autocorrelation coefficient values for the two voxels (Kaneoke et al., 2012). Voxel‐wise mean *Z* values across sessions were used to produce three adjacency matrixes with 1 for an edge or connection and 0 for no connection for each participant with a threshold of *Z* = 1.96 (*p* = .05), *Z* = 2.58 (*p* = .01), and *Z* = 3.28 (*p* = .001).

We used nAC, which was recently proposed by Ghosh and Lerman (Ghosh & Lerman, 2011), to identify local and global network properties by varying the attenuation parameter (α). We calculated nAC with two values, α = 0 and 1/λ_1_ (λ_1 _= maximum eigenvalue of the adjacency matrix), which are called nAC_0_ and nAC_1_, respectively, in this study. nAC_0_ is related to the *degree centrality* (Bullmore & Sporns, 2009) and reflects local (directly connected) network properties. In contrast, nAC_1_ is related to the *eigenvector centrality* (Lohmann et al., 2010), and the value reflects the relationship between the node and the entire network structure.

Note that we did not set the same total number of *edges* for each participant, although this is necessary to assess network structures, such as clustering, path length, and the small world index (Fornito, Zalesky, & Breakspear, 2013). Instead, in this study, we considered the number of edges in each brain as representative as the participant's individuality. The number of edges for each participant was counted, and we checked for any differences when the two groups were compared, such as between males and females. Furthermore, we investigated the effects of edge numbers on the network properties at each node. For each participant, we measured the mean nAC_0_ and nAC_1_ values at each brain region (see below) and investigated the relationship between these values and the number of edges for males and females.

### 2.8 Estimation of brain network properties at each node

**Figure 2:**
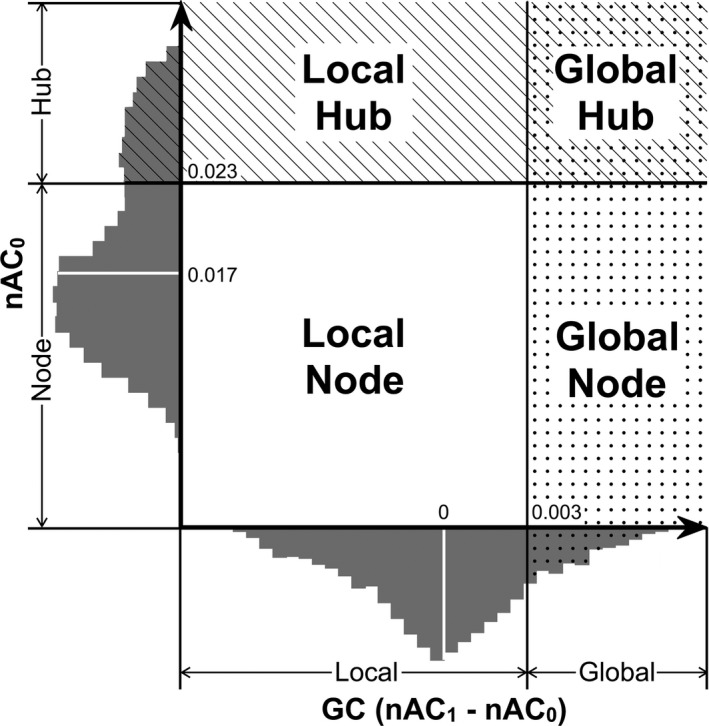
Classification of brain network nodes. Each node was classified into four types based on the values of nAC_0_ and GC (nAC_1_–nAC_0_). The *x*‐axis shows the distribution of GC and the mean value (0.0) with a white line, and 1 *SD* value (0.003) with a black line. The *y*‐axis shows the distribution of nAC_0_ and the mean value (0.017) with a white line and 1 *SD* value (0.023) with a black line

One of the best advantages of using the nAC in the evaluation of brain functional connectivity is that the values of nAC_0_ (which stands for local connectivity strength) and nAC_1_ (which stands for eigenvector centrality) at each node can be directly compared (Ghosh & Lerman, 2011). Thus, we measured the difference between these two values (nAC_1_–nAC_0_) at each node and referred to the results as GC. High values for nAC_0_ indicate that the node is locally important in that the node directly connects with many nodes in a local community. The value of nAC_1_ indicates the magnitude of connectivity within a whole network. Thus, positive GC means that the node is more globally important than locally important (in its local community). Using GC and nAC_0_, we classified each node into four different types (global hub, global node, local hub, and local node; Figure 2) based on the distribution of the two values in both males and females: A node was classified as a “hub” when the value of nAC_0_ was higher than the mean + *SD*; otherwise, it was classified as a “node.” Furthermore, the node and hub were classified by the value of GC as “global” (when its GC was higher than the mean + *SD*) or “local” (when its GC was lower than the mean + *SD*). Note that the mean + *SD* of GC was a positive value because the mean value was 0.0 (see Results). For example, a node was classified as a “global hub” when its nAC_0_ was higher than the mean + *SD* and GC was also higher than the mean + *SD* of the distribution, indicating that the node was both locally and globally important. By contrast, a “global node” does not often directly connect to other nodes but connects with globally important nodes.

### 2.9 Statistical analysis

To assess the statistical significance of gender differences, we first measured the mean network properties at each node for males and females. Based on the mean values of nAC_0_ and nAC_1_ for males and females at each node, these nodes were classified into four types as described above (see Figure 2). Then, we measured the percentage of each type of node among the tonal nodes at each region of the gray matter. The gray matter was classified into the following regions based on the AAL: ventral frontal (FRv), medial frontal (FRm), lateral frontal (FRl), sensorimotor (SM), cingulate (CIN), lateral parietal (PAl), medial parietal (PAm), insula (INS), limbic (LIM), temporal (TE), occipital (OC), cerebellar (CER), BG, and TH. The classification details are shown in Table S1. We then used a chi‐square test to assess the statistical significance of the difference in the distribution of each node type in 14 regions between males and females. Because we evaluated four node property distributions, *p* values were corrected for multiple comparisons with Bonferroni's method. A statistically significant difference in the distribution (the percentage of each node properties in each region) suggests that the male brain data were extracted from a population that was different from the population of female brain data. Furthermore, we performed a permutation test to validate the difference in node property proportions in each region between males and females. First, 200 participants’ data were randomly divided into two groups (100 participants in each group), and the mean nAC_0_ and nAC_1_ values were calculated for each node to determine the node property for each node. The proportion of node types in each region was then measured, and the difference between the two groups was calculated. This procedure was repeated 10,000 times, and the number of trials (*n*) showing a difference exceeding the original data (the proportion of the node type in a region for males minus that for females) was counted to determine the “permutation *p* value” (*n*/10,000).

Similarly, we investigated the effect of the digit ratio and menstrual phase on the distribution of the node types.

## 3 RESULTS

The mean age and head motion parameter (mean Power's FD) were not significantly different between males and females (Table 1). Although all of the participants were right‐handed (Edinburgh handedness score >70), the female score was significantly larger than that for males (*p* < .05, Mann–Whitney *U* test). The mean digit ratio for males (0.938 ± 0.026) was significantly lower than that for females (0.951 ± 0.030) (Table 1). Gray‐matter volume was significantly larger for males than for females, but the ratio to TIV was larger for females than for males, consistent with a previous study (Chen et al., 2007; Gur et al., 1999).

Table 2 shows the mean digit ratios for the subgroups divided by the mean digit ratios and menstrual phase. Mental activity during MRI acquisition was not significantly different between the two groups (*p* > .05, chi‐square test; Figure S1). The excluded sessions were not significantly different between the two groups (Table S2). The results for the different thresholds for adjacency matrix and preprocessing 2 are shown in Supporting Information.

The mean (±*SD*) values for nAC_0_, nAC_1_, and GC from each dataset are shown in Table 3. We first describe the results for preprocessing 1 and the threshold of *Z* = 2.58 for an adjacency matrix (see Materials and Methods) and then the results for the other datasets in the Supporting Information. The mean nAC_0_ value was 0.0169 ± 0.006, and the mean GC value was 0.00 ± 0.0031 for this dataset. Table 4 shows the mean number of edges for the male and female groups and their subgroups. The mean number of edges for females (10.2 × 10^5 ^± 2.38 × 10^5^) was significantly larger than that for males (9.52 × 10^5^ ± 2.18 × 10^5^) (*p* = .042, *t* test).

**Table 4:** Number of edges

	Malemean ± *SD *× 10^5^ (*N*)	^*p*^	Femalemean ± *SD* ×10^5^ (*N*)	*p*	FOLmean ± *SD* ×10^5^ (*N*)	LUTmean ± *SD *× 10^5^ (*N*)	*p*
ALL	9.52 ± 2.18 (100)		10.2 ± 2.38 (100)	.042^a^	9.94 ± 2.33 (53)	10.4 ± 2.11 (40)	.341^b^
High 2D:4D	9.42 ± 2.22 (53)	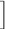 .624^c^	10.4 ± 2.53 (51)	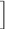 .343^c^	10.3 ± 2.53 (26)	10.3 ± 1.88 (22)	.967^b^
Low 2D:4D	9.64 ± 2.16 (47)		9.95 ± 2.21 (49)		9.62 ± 2.12 (27)	10.5 ± 2.41 (18)	.204^b^

*p* values by *t* test ^a^between males and females, ^b^between follicular phase and luteal phase females, ^c^between high and low digit groups. FOL, follicular phase; LUT, luteal phase.

### 3.1 Gender differences in the distribution of the network properties

**Figure 3:**
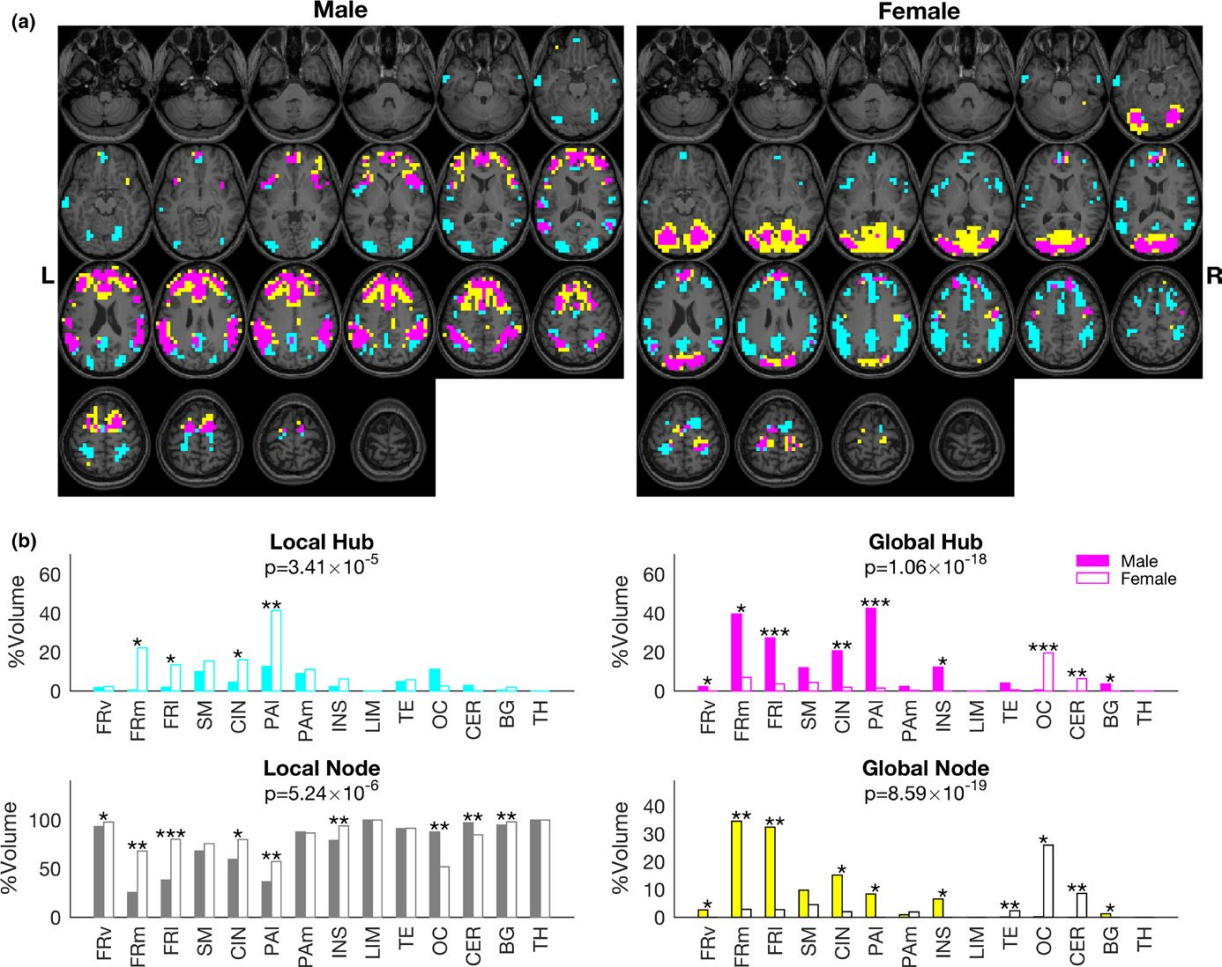
Distribution of three node types in male and female brains. (a) For each male and female group, we calculated the mean nAC_0_ and nAC_1_ values for each node to determine the node type using the data for an adjacency matrix with a threshold of *Z* = 2.58 and using data from preprocessing 1. For the male brain, global hubs (magenta voxels) and global nodes (yellow voxels) were distributed in the frontal, cingulate, and parietal areas. By contrast, these hubs were more dominant in the occipital and cerebellar regions in the female brain. Cyan voxels: local hubs. Local nodes are not shown. (b) The percentages of global hubs, global nodes, local hubs, and local nodes in the total voxels in each region for male and female groups are shown with separate graphs. A chi‐square test revealed that the all four node type distributions were significantly different between males and females (*p* values corrected with Bonferroni's method are shown in each graph). A permutation test showed significant differences in the percentage of node types across the different regions as shown by asterisks: *p < .05; **p < .01; ***p < .001. Closed columns: male; open columns: females. FRv, ventral frontal; FRm, medial frontal; FRl, lateral frontal; SM, sensorimotor; CIN, cingulate; PAl, lateral parietal; PAm, medial parietal; INS, insular; LIM, limbic; TE, temporal; OC, occipital; CER, cerebellar; BG, basal ganglia; TH, thalamus

Figure 3a shows the distribution of the three node types for males and females. Global nodes were mainly distributed in the frontal and TE areas for males and in the OC and CER areas for females. The percentage of each node type in the 14 regions is shown in Figure 3b. A chi‐square test revealed that the distributions of the four node types for males were significantly different from those for females (*p* < .00001, chi‐square test). Moreover, the permutation test revealed that the percentages of global hubs and global nodes in the three frontal regions, CIN, PAl, INS, and BG for males were significantly larger than those for females (*p* < .05, permutation test). By contrast, the percentages of global hubs and global nodes in the OC and CER regions for females were significantly larger than those for males (*p* < .05, permutation test).

Because the mean handedness score for males was significantly lower than that for females (Table 1), we checked the network property difference between males and females using data from participants whose handedness scores were 100 (38 males and 57 females). Figure S2 shows that the distribution differences of the network properties in each region were remarkably similar to those for the data from all participants (Figure 3b), especially for global hubs and global nodes. A permutation test revealed that the percentages of global hubs and global nodes in the PAl region for males were significantly larger than those for females (*p* < .01 and *p* < .05, respectively). By contrast, the percentages of global hubs in the OC and CER regions for females were significantly larger than those for males (*p* < .001 and *p* < .01, respectively).

The relationship between the gray‐matter volume/ratio and network properties (nAC_0_ and nAC_1_) for each participant in each region is shown in Figure S3 and S4. There was no significant relationship between these values in all regions (*p* > .05, Pearson's methods). The relationship between the number of edges and network properties for each subject at each region is shown in Table 5 and Figure S5. This relationship varied with both region and gender; in the FRm region, the number of edges was negatively related to the mean nAC_0_ and nAC_1_ for females but not for males. Furthermore, in the INS region, we detected a positive relationship for females but not for males.

**Table 5:** Correlation between the number of edges and network properties in each region

	nAC_0_		nAC_1_	
	Male	Female	Male	Female
FRv	–	–	–	–
FRm	–	−0.336 (6.38 × 10^−4^)		−0.232 (0.0200)
FRl	–	–	–	–
SM	–	–	–	+0.282 (4.50 × 10^−3^)
CIN	–	–	–	–
PAl	−0.359 (0.00024)	−0.444 (3.62 × 10^−6^)	–	−0.235 (0.0185)
PAm	−0.318 (0.00126)	−0.219 (0.0283)	–	–
INS	–	+0.467 (9.65 × 10^−7^)	–	+0.354 (3.08 × 10^−4^)
LIM	+0.394 (5.08 × 10^−5^)	+0.475 (6.04 × 10^−7^)	+0.450 (2.60 × 10^−6^)	+0.547 (3.94 × 10^−9^)
TE	–	–	–	–
OC	–	–	–	–
CER	+0.311 (0.00162)	+0.199 (0.0468)	+0.203 (0.0428)	–
BG	+0.465 (1.12 × 10^−6^)	+0.544 (4.90 × 10^−9^)	+0.260 (9.11 × 10^−3^)	+0.408 (2.51 × 10^−5^)
TH	+0.565 (9.37 × 10^−10^)	+0.514 (4.47 × 10^−8^)	+0.427 (9.65 × 10^−6^)	+0.560 (1.33 × 10^−9^)

Values are presented as Pearson's correlation coefficients between the number of edges and mean nAC_0_/nAC_1_ in each region for each participant with uncorrected *p* values in parentheses. Values are omitted when *p *≧ .05. See the Figure 3 legend for region abbreviations.

### 3.2 Effect of the digit ratio on the distribution of the network properties

**Figure 4:**
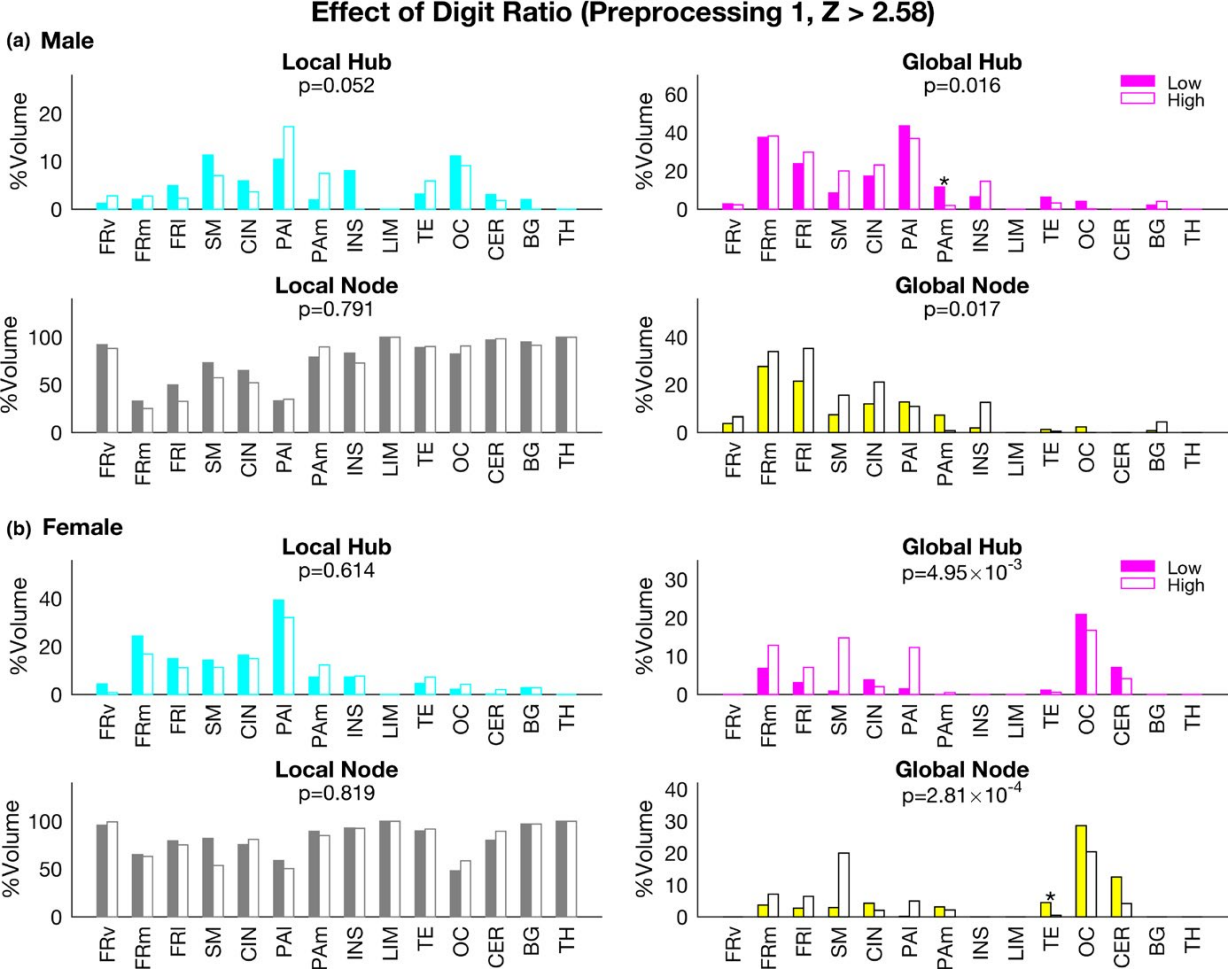
Effects of the digit ratio on brain network properties. The percentage of each node types in each region for males (top) and for females (bottom) is shown as in Figure 3. Chi‐square test results are shown by *p* values (corrected with Bonferroni's method) in each graph. The results from a permutation test are also shown by an asterisk (*p < .05). Closed columns: low digit ratio group; open columns: high digit ratio group

The male and female groups were further divided into two subgroups based on their digit ratios (Table 2). For males, the distributions of node types were significantly different between the two subgroups in terms of global hubs and global nodes (chi‐square test, *p* = .016 and .017, respectively; Figure 4a). A permutation test revealed that the percentage of global hubs in the PAm region of the low digit ratio group was significantly higher than that in the high digit ratio group (*p* = .032, permutation test).

For females, the distributions of node types were significantly different between the two subgroups in terms of global hubs and global nodes (chi‐square test, *p* = .005 and 2.81 × 10^−4^, respectively; Figure 4b). A permutation test revealed that the percentage of global nodes in the TE region for low digit ratio group was significantly higher than that for the high digit ratio group (*p* = .047, permutation test).

**Figure 5:**
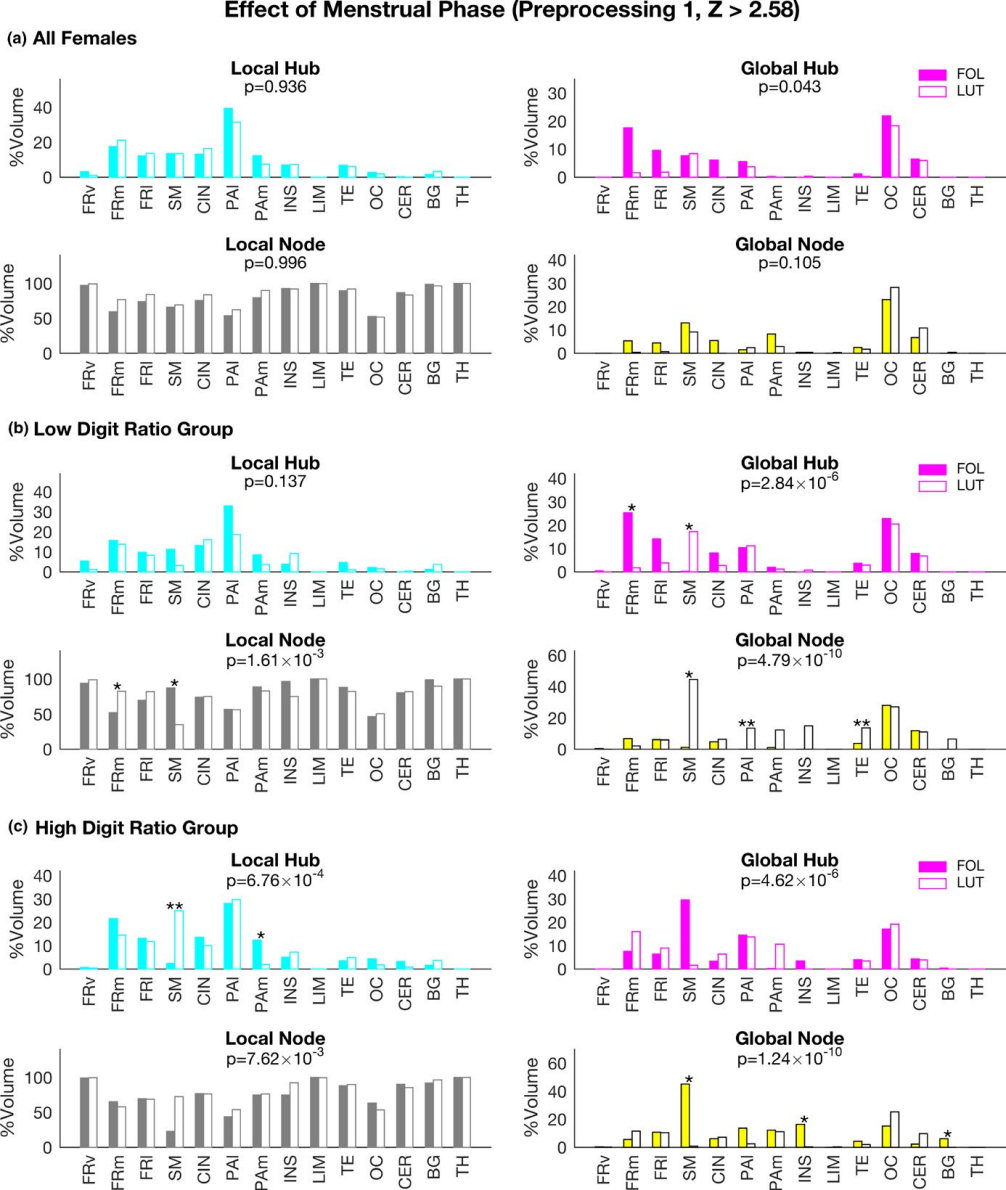
Effects of menstrual phase on network properties. The percentage of each node type in each region is shown as in Figure 3. (a) The results for the data for 47 follicular phase and 46 luteal phase females. (b) The results for the low digit ratio group. (c) The results for the high digit ratio group. Chi‐square test results are shown by a *p* value (corrected with Bonferroni's method) in each graph. Permutation test results are shown by asterisks (*p < .05; **p < .01). Closed columns: follicular phase group; open columns: luteal phase group. FOL, follicular phase; LUT, luteal phase

To investigate the effect of the digit ratio on the differences in network properties caused by the menstrual cycle, we first compared node type distributions between the follicular and luteal phases. When the effect of menstrual phase was assessed by a permutation test using data from 93 females, we did not find any significant difference, although a chi‐square test revealed that the global hub distributions were affected the by the menstrual phases (Figure 5a). Next, we assessed the effects of menstrual phase using two further subgroups (one for low digit ratios and another for high digit ratios). For the low digit ratio group, menstrual phase affected the distribution of global hubs, global nodes, and local nodes (Figure 5b). A permutation test revealed that the percentage of global hubs in the FRm region for the follicular phase group was significantly higher than that for the luteal phase group (*p* = .016, permutation test). By contrast, the percentage of global hubs in the SM region for the luteal phase group was significantly higher than that for the follicular phase group (*p* = .049, permutation test). For the high digit ratio group, menstrual phase significantly affected the distribution of the four node types (Figure 5c). A permutation test revealed that the percentages of global nodes in the SM, INS, and BG regions for the follicular phase group were significantly higher than those for the luteal phase group (*p* < .05, permutation test).

## 4 DISCUSSION

Gender (male and female), the 2D:4D digit ratio (low and high digit ratios), and menstrual phase (follicular and luteal phases) all showed statistically significant differences in the distribution of brain network properties across several brain regions, even though there were some differences in our results due to the preprocessing of rs‐fMRI data.

### 4.1 Effects of gender on the brain networks

Most brain regions, including the subcortical regions such as the BG and the CER, showed gender differences in the distribution of network properties except the PAm, SM, and LIM regions, and the TM. On average, males had a higher percentage of global hubs and global nodes in the frontal, CIN, and PAl regions; by contrast, females showed higher percentages of these nodes in the OC and CER regions than did males (Figure 3). Similar results were obtained for the other datasets with different threshold *Z* values that were used to create an adjacency matrix (Figure S6) and with different preprocessing strategies to reduce nuisance noise (Figure S7). Consequently, these results suggest that brain network properties in males constitute a distinct population compared with those in females. These results may agree with a previous analysis of functional connectivity that showed a higher number of connections between modules in males than in females (Satterthwaite et al., 2015), because we found a higher number of global hubs in males than in females (see Figure 3a). That is, because global hubs are by definition expected to have a larger number of connections with other communities than with local nodes.

Global signal regression increased the detectability of gender differences in the network properties of our data. When global signal regression and head motion regression were omitted during the preprocessing stage, a permutation test detected significant differences in the percentages of node types in the nine regions (Figure S7). The number of regions increased to 32 regions (Figure 3) with the global signal and head motion regressions (preprocessing 1). We considered that at least for our data, global signal (and head motion) regression increased sensitivity to distinguish gender differences in the network properties, because nonsignificant differences with a similar tendency were found for most regions using data from preprocessing 2 (no global signal and head motion regressions; Figure S7): Percentage of global hubs and global nodes in the frontal and lateral parietal regions for males are larger than those for females. By contrast, female dominance of the global hubs and global nodes in the OC and CER regions were found only for the data from preprocessing 1. When data from preprocessing 2 were used, almost all nodes in these regions were identified as local nodes (Figure S7).

Previous whole‐brain functional connectivity studies in a relatively large number of participants revealed various differences in terms of network organizations between males and females (Allen et al., 2011; Biswal et al., 2010; Filippi et al., 2013; Jung et al., 2015; Satterthwaite et al., 2015; Scheinost et al., 2015; Tomasi & Volkow, 2012; Zhang et al., 2016), although some studies did not (Bluhm et al., 2008; Weissman‐Fogel, Moayedi, Taylor, Pope, & Davis, 2010). These findings suggest the existence of gender differences in the functional network organization of the brain but also indicate that these differences are very subtle and difficult to distinguish. As shown in Figure S5, the distributions overlap too much to support a dimorphic view of gender‐related differences in brain function (Joel et al., 2015). These inconsistent differences (brain regions and functional connectivity strength) between males and females could also be due to the functional connectivity parameters being measured and the preprocessing methods utilized. Thus, it is difficult to compare our present results with those of previous studies because we evaluated both local (nAC_0_) and global (nAC_1_) functional connectivity strength at each node for the first time. However, our present study showed the distinct differences in the population of the network properties of the brain between males and females even after matching for age, educational level, handedness, MRI acquisition time and MRI equipment.

Gray‐matter volume does not appear to affect our network property analysis because there was no significant relationship between the gray‐matter volume/ratio and network properties (Figures S3 and S4). The mean number of edges for females was significantly larger than that for males (Table 4), which is consistent with a previous structural connectivity study (Szalkai, Varga, & Grolmusz, 2015). However, this finding cannot account for the difference in the brain network properties between males and females for the following reasons: First, the number of edges was not simply related to network properties; the relationship varied among regions and with gender (Table 5 and Figure S5). Second, our results for other datasets (preprocessing 1 and threshold *Z* = 1.96, and preprocessing 2 and threshold *Z* = 2.58) also showed similar gender differences in network properties, even though the mean number of edges did not differ between males and females (Figure S6a and S7).

Most likely, functional brain network changes with the menstrual cycle in females cannot account for the distinct differences between males and females. Although we found significant difference in the distributions of global hubs between the follicular and luteal phases (*p* = .043 corrected for multiple comparison with Bonferroni's method, chi‐square test; Figure 5), the significance level was marginal, and the permutation test did not find any significant differences in the percentage of global hubs between the two phases, suggesting that the effects of the menstrual cycle were not be so large as the effects of gender differences.

Interestingly, mental activity during MRI acquisition did not differ between males and females, despite the different brain network properties. Mental activity at rest is likely related to a default network (Buckner, Andrews‐Hanna, & Schacter, 2008; Kucyi & Davis, 2014; Mason et al., 2007) and its interaction with other networks (Doucet et al., 2012). Moreover, gender differences in the default network have been reported (Biswal et al., 2010; Jung et al., 2015). Our results also suggest a default network difference between males and females because we found differences in the percentages of global hubs within the cardinal regions for the default network such as the FRm and PAl regions (Figure 3a). One possibility is that the ReSQ could not detect gender differences because of the simple classification of its contents. Another possibility is that the gender differences in brain network properties developed in a manner that achieves similar behavioral and cognitive outcomes by compensating for the effects of sex hormone levels, which may lead to the undesirable dimorphic gender differences as proposed by De Vries (De Vries, 2004).

### 4.2 Effects of the digit ratio on the brain network properties

The 2D:4D digit ratio is related to prenatal sex hormone levels (Manning et al., 2003, 2014). For the first time, our present study revealed that the digit ratio differentially affects the brain networks of adult males and females. In particular, the percentage of global hubs in the PAm region for the low digit ratio group of males was significantly higher than that for the high digit ratio group. By contrast, the percentage of global nodes for the female low digit ratio group in the TE region was significantly higher than that for the high digit ratio group. The PAm region includes the posterior cingulate cortex (PCC) and precuneus, which is the core of the default network (Greicius, Krasnow, Reiss, & Menon, 2003) and is related to various cognitive tasks (Utevsky, Smith, & Huettel, 2014). A recent study showed that the connectivity (degree centrality) of the PCC in the resting state is related to the speed of task performance (Lin et al., 2016). The task performance of financial traders is inversely related to the digit ratio (Coates, Gurnell, & Rustichini, 2009). Thus, our finding that the low digit ratio group had higher percentage of global hubs in the PAm region than did the high digit ratio group may indicate the neural correlate of task performance of financial traders. However, these results varied among different preprocessing strategies (see Figures 4 and S8). These differences might be due to the noise that could not be removed in preprocessing 2 as seen for the other results.

### 4.3 Effects of menstrual phase on the brain network properties

In females, the menstrual cycle influences brain activity (Sundstrom Poromaa & Gingnell, 2014; Thimm et al., 2014; Weis et al., 2008; Zhu et al., 2015) and is suggested to influence brain functional connectivity (Arelin et al., 2015; Weis et al., 2011) via levels of sex hormones such as progesterone and estrogen. The reason why several studies did not show any difference in resting‐state functional connectivity across menstrual phases (De Bondt et al., 2015; Hjelmervik, Hausmann, Osnes, Westerhausen, & Specht, 2014; Syan et al., 2017) could be due to an insufficient number of participants to reveal subtle changes in the brain functional connectivity.

The effects of the menstrual cycle on the brain network properties might be different between the low and high digit ratio groups, because a permutation test more often revealed differences in brain regions (five regions) in the low digit ratio groups than in the high digit ratio group (three regions) for the global hubs and global nodes in a different manner (Figure 5). The results may be related to the previous our study that showed women with a higher right 2D:4D ratio had lower premenstrual symptom severity (Kaneoke et al., 2017). Similar results were observed for the data with preprocessing 2 (Figure S9). Furthermore, a recent study showed that the effects of testosterone administration on female brain functional connectivity vary with digit ratios (Chen et al., 2016), which suggests that the prenatal sex hormone exposure affects female brain functional changes with sex hormones in adulthood.

### 4.4 Limitations

Here, we note several limitations of the results in this study. We used the 2D:4D digit ratio for the estimation of prenatal sex hormones exposure. Although the prenatal sex hormones causally affect the 2D:4D ratio in animal experiments (Zheng & Cohn, 2011), and substantial evidence supports the relationship between prenatal sex hormones levels and the 2D:4D ratio (Manning et al., 2014), the 2D:4D ratio is also affected by several other factors, such as genetic influences and environmental factors (Hiraishi, Sasaki, Shikishima, & Ando, 2012). Further, the effects of the 2D:4D ratio on the brain network were investigated by the groups classified by the median 2D:4D ratio, which depends on the population studied and does not provide information regarding the amount of prenatal sex hormones. Thus, the results could potentially vary to some extent based on the population studied.

Second, it should be noted that we estimated the menstrual phase by the last menstrual date; we did not measure the levels of sex hormones at the time of the experiment. Estrogen levels markedly changes in the follicular phase, and the functional connectivity in specific regions changes with estrogen levels (Engman, Linnman, Van Dijk, & Milad, 2016; Lisofsky et al., 2015). Thus, the effects of menstrual phase on brain network properties must be more precisely evaluated with sex hormone levels in future studies.

Third, according to our recent study, females with a lower digit ratio of the right hand tend to have greater premenstrual symptoms (Kaneoke et al., 2017). Thus, our low digit ratio group may have more participants with premenstrual syndrome and premenstrual dysphoric disorder than the high digit ratio group, which could affect the results (Comasco & Sundstrom‐Poromaa, 2015). Mental states for both male and female participants were not evaluated to examine the effects of psychiatric disorders on the functional connectivity. However, it is unlikely that students in a depressive state would come to an unfamiliar institute to participate in an experiment.

## 5 CONCLUSION

In conclusion, this study showed that the distribution of brain network properties, as determined by nAC, was different between males and females, even though some results varied due to differences in rs‐fMRI data processing. The 2D:4D digit ratio differentially affected the brain network property distributions of both males and females. In females, menstrual phase affected the brain network property distribution, which varied with the 2D:4D digit ratio. These results suggest that the functional brain network properties vary with gender, prenatal sex hormone exposure, and the menstrual cycle. These factors might be important for elucidating the fundamental mechanisms underlying how gender differences occur in brain disorders and how sex hormones affect their prevalence and symptoms.

## CONFLICT OF INTERESTS

The authors declare no conflicts of interest.

## AUTHORS’ CONTRIBUTIONS

TD performed the experiments, analyzed the data, and created the figures; MT performed the experiments; and YK conceived and designed the experiments, and wrote the manuscript.
