# Supplementary material for: Effects of gender, digit ratio, and menstrual cycle on intrinsic brain functional connectivity: A whole‐brain, voxel‐wise exploratory study using simultaneous local and global functional connectivity mapping
Source: Brain Behav. 2017 Dec 19;8(1):e00890. doi: 10.1002/brb3.890 (PMC5853634; doi:10.1002/brb3.890)
Supplement: Supplementary file 1 [file BRB3-8-e00890-s001.pdf]

Supplementary Results

Effects of threshold difference upon the network properties

When an adjacency matrix was created with a threshold of Z = 1.96, the mean number of edges for males ($1.67x10^6 \pm 0.32x10^6$) was not significantly different from that for females ($1.76x10^6 \pm 0.35x10^6$) (p>0.05, t-test). In contrast, the mean edge number for females ($5.59x10^5 \pm 1.45x10^5$) was significantly larger than that for males ($5.18x10^5 \pm 1.31x10^5$) (p = 0.036, t-test) when an adjacency matrix was created with the threshold of Z=3.28.

For the data created with a threshold of Z=1.96, the distributions of global hubs, global nodes and local nodes for males were significantly different from those for females (Chi-square test, p = 0.034, $3.9x10^{-6}$, and 0.0013, respectively) (**Figure S6A**). The data created with a threshold of Z=3.28 also showed significant difference in the distribution of global hubs between males and females (p = $4.95x10^{-5}$) (**Figure S6B**).

Effects of preprocessing upon the network properties

This data set was used to investigate the effect of preprocessing upon the results, because it is, in theory, impossible to discriminate signals related solely to the brain activity from the functional image data (Bright and Murphy, 2015; Pujol, et al., 2014) and there is no standard preprocessing method for the functional images (Liu, 2016). In particular, global signals could convey brain activity information, and its regression away from the data could cause artificial noise (Liu, et al., 2017), though global signal regression has been shown to be effective in removing nuisance noise related to head motion, cardiac pulsation, and respiration (Power, et al., 2016).

When an adjacency matrix was generated with a threshold of 2.56 using the data from preprocessing 2 (CompCor without global signal head motion regression), the mean number of edges for males ($1.76x10^6 \pm 0.67\ x10^6$) was not significantly different from that for females ($1.86x10^6 \pm 0.65\ x10^6$) (p = 0.31, t-test). The distributions of the global hubs and global nodes in the 14 regions were significantly different between male and female groups (Chi-square test, p = 0.009 and $1.13x10^{-5}$) (**Figure S7**) as seen for the data from 'preprocessing 1' (**Fig. 3**). In other words, the percentages of global hubs in the three frontal regions, the cingulate, and the insula for males were significantly higher than those for females; observations which were validated by the

permutation test ($p<0.05$). There were no significant differences in the distributions of local nodes and local hubs in contrast to the data from ‘preprocessing 1’ which showed significant differences among these distributions.

The effects of digit ratios upon the distributions of node types were shown to be significant for both males (**Figure S8A**) and females (**Figure S8B**). The permutation test revealed that the percentage of global nodes for high digit ratio males in the sensorimotor region was significantly higher than that for low digit ratio males. For females, the percentage of global nodes in the lateral parietal and occipital regions were significantly different when compared high and low digit ratio subgroups.

For the female low digit ratio group, we found that menstrual phase cause significant effects upon the distributions of global hubs, global nodes, and local nodes. as revealed by Chi-square tests. Several of these regional differences were further validated by the permutation test (**Figure S9A**). Menstrual phase also affected the global node distribution for the high digit ratio group (**Figure S9B**). The permutation test revealed that the percentage of global nodes in the medial parietal region for the luteal phase group was significantly higher than that for the follicular phase group ($p = 0.034$, permutation test).

Table S1. Regions determined with AAL

Region	Abbreviation	AAL Nomenclature*
Ventral Frontal	FRv	Olfactory<br>Frontal Inf/Sup/Med/Mid Orb<br>Rectus
Medial Frontal	FRm	Frontal Sup Medial<br>Frontal Mid
Lateral Frontal	FRl	Frontal Inf Tri/Oper<br>Frontal Sup
Sensorimotor	SM	Precentral<br>Supp Motor Area<br>Rolandic Oper<br>Postcentral
Cingulate	CIN	Cingulum Ant/Mid
Lateral Parietal	PAl	Parietal Sup/Inf<br>SupraMarginal<br>Angular
Medial Parietal	PAm	Precuneus<br>Paracentral Lobule<br>Cingulum Post
Insula	INS	Insula
Limbic	LIM	Hippocampus<br>Parahippocampal<br>Amygdala
Temporal	TE	Heschl<br>Temporal Pole Mid/Sup<br>Temporal Sup/Mid/Inf
Occipital	OC	Calcarine<br>Cuneus<br>Lingual<br>Occipital Sup/Mid/Inf<br>Fusiform
Cerebellum	CER	Cerebellum Crus1/2<br>Cerebellum 3/4/5/6/7b/8/9/10<br>Vermis 1-10
Basal Ganglia	BG	Caudate<br>Putamen<br>Pallidum
Thalamus	TH	Thalamus

* Tzourio-Mazoyer et al., NeuroImage 15, 273-289 (2002).

Table S2. Number of participants with excluded rs-fMRI sessions

Number of sessions excluded	0	1	2	3
Male	119	14	4	0
Female	100	13	4	0

Figure S1. Mental activity during MRI acquisition

Results of the Resting-state questionnaire (ReSQ) are shown for males and females. **A-E** show the proportion (%) of participants spending time on each imagination type, as shown in the title. **F** shows the number of participants who prioritized their time on each imagination type (more than 50% of time). There were no significant differences in the distributions by Chi-square test (p>0.05). IMAG: visual mental imagery; LANG: inner language and auditory mental imagery; SOMA: somatosensory awareness; MUSI: inner musical experience; NUMB: mental manipulation of numbers.

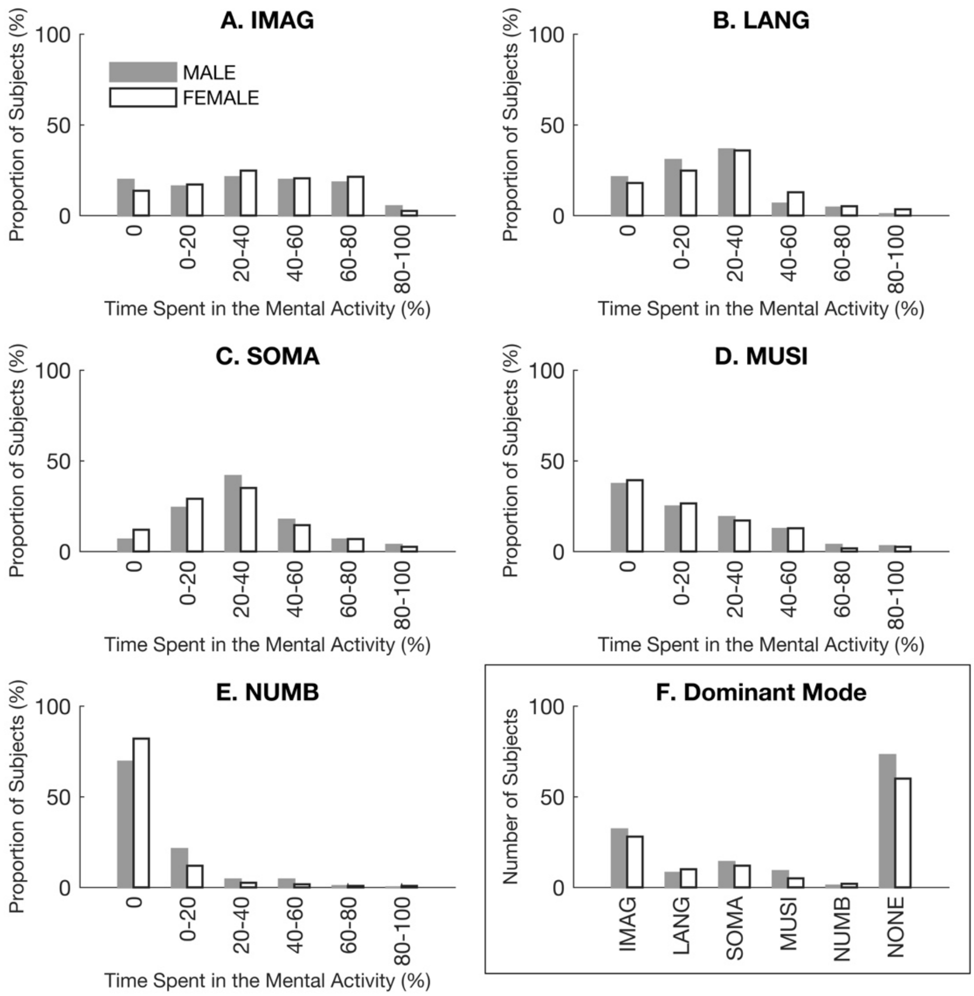

Figure S2. Gender differences in the network properties for the participants with a handedness score of 100

Gender difference in node type distributions were investigated using data from participants whose handedness scores were 100 (38 males and 57 females). The percentage of each node types in each region are shown as in **Fig. 3**. The results are similar to those shown in **Fig. 3**. *p<0.05 and *p<0.01 by permutation test. Chi-square test results are shown by p values (corrected with Bonferroni method) in each graph.

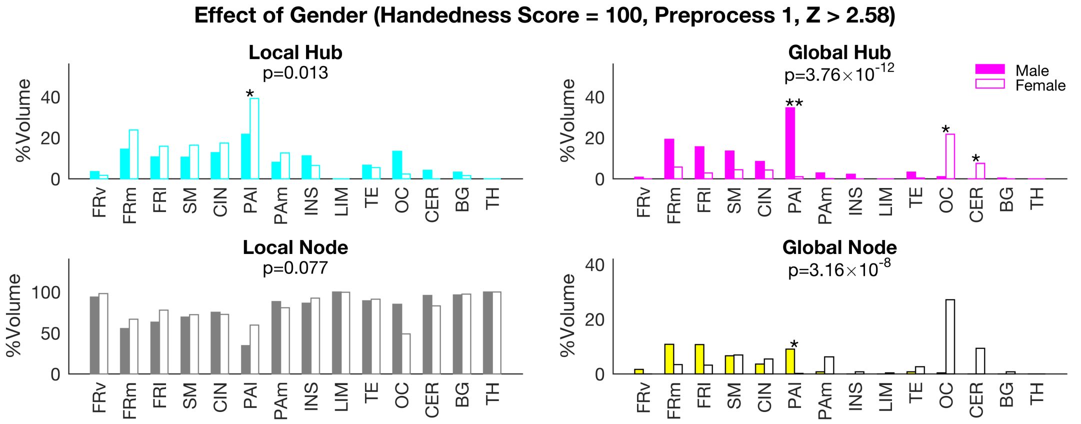

Figure S3. Relationship between Gray matter volume and network properties.
The gray matter (GM) volume (x axis) for each participant and the mean nAC_0 (top) and nAC_1 (bottom) in each region (see **Table S1**) for each participant were plotted with regression lines. There was no significant relationship between them at all the regions ($p>0.05$, Pearson's method) for both males and females.

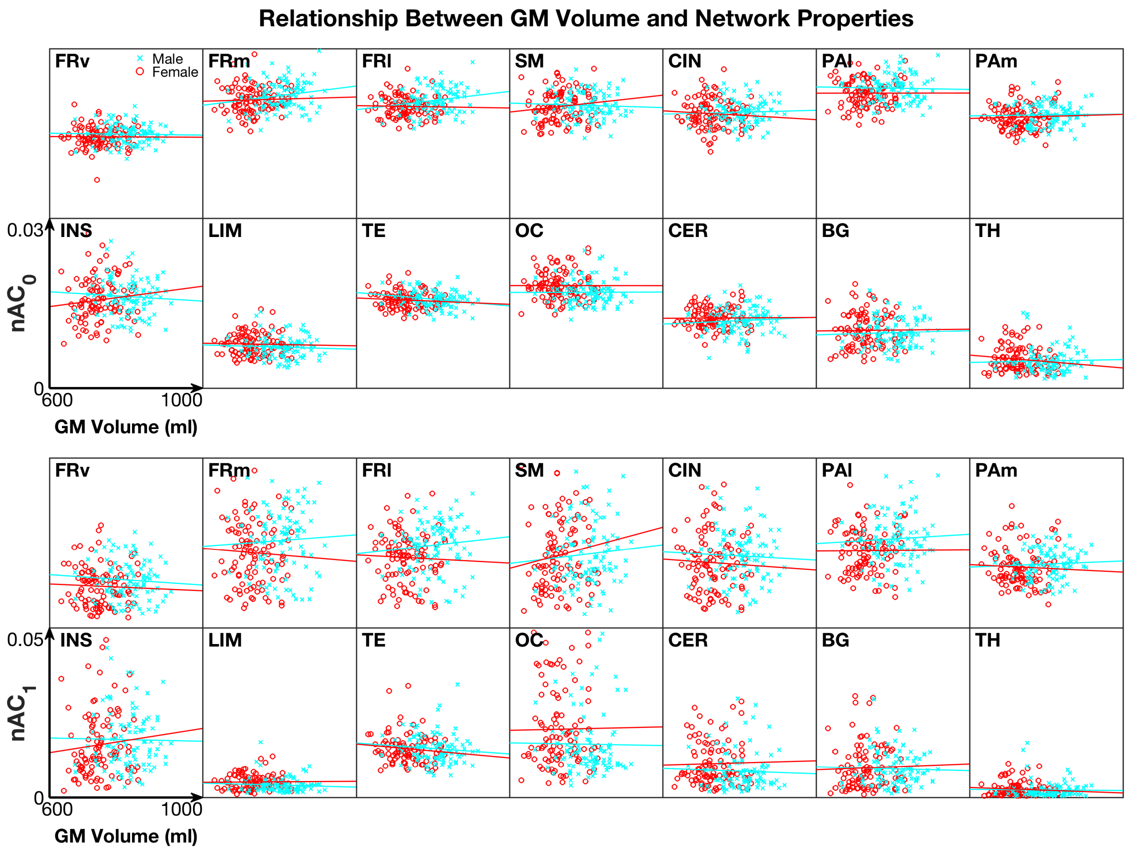

Figure S4. Relationship between Gray matter volume and network properties. The gray matter (GM) ratios to total intracranial volume (TIV) (x axis) for each participant and the mean nAC_0 (top) and nAC_1 (bottom) in each region (see **Table S1**) for each participant were plotted with regression lines. There was no significant relationship between them at all the regions ($p>0.05$, Pearson's method) for both males and females.

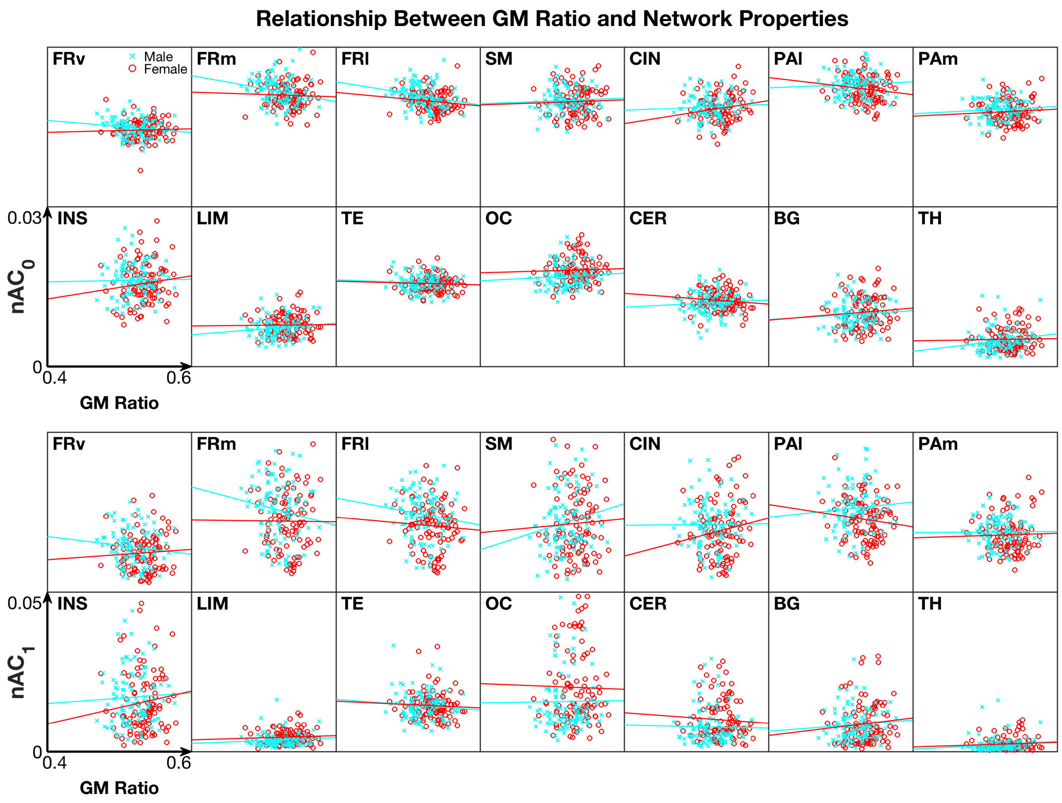

Figure S5. Relationship between the number of edges and nAC_0/nAC_1

The number of edges (x axis) for each participant and the mean nAC0 (top) and nAC1 (bottom) in each region (see **Table S1**) for each participant were plotted with regression lines. See **Table 5** for Pearson's correlation coefficients and p values.

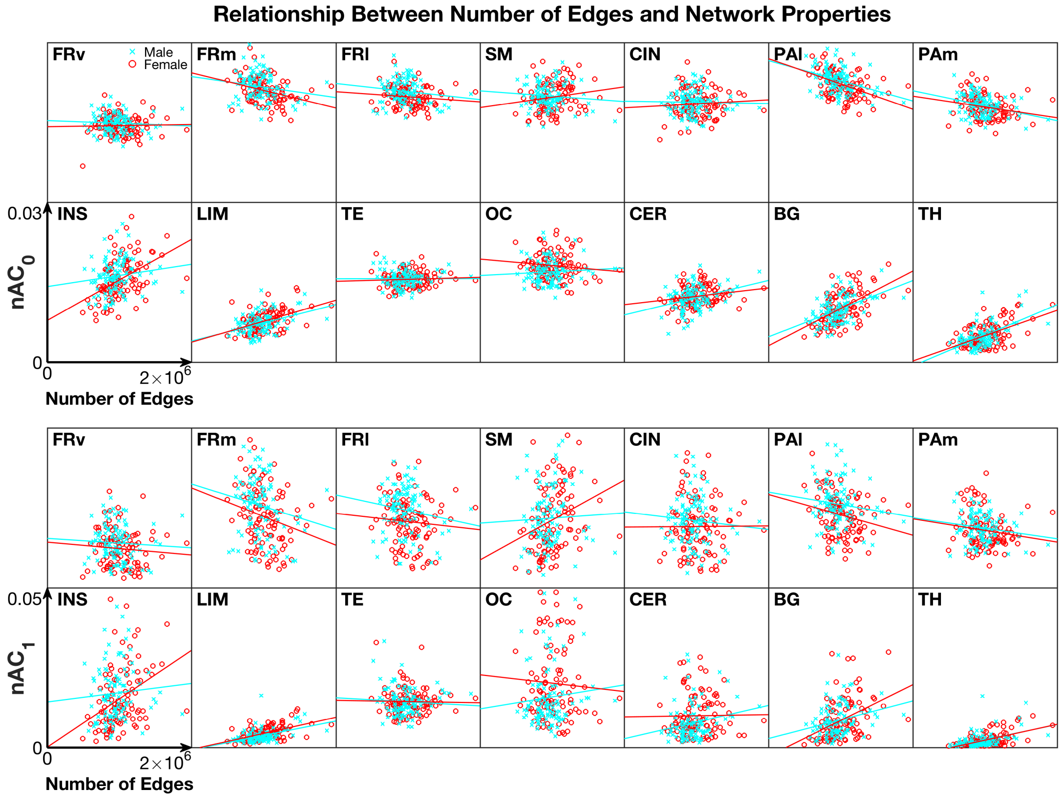

Figure S6. The effect of threshold in creating an adjacency matrix upon network variation with gender.

Gender difference of the node type distributions were investigated using the data from preprocessing 1 and with a threshold of Z = 1.96 (top) and 3.28 (bottom) for an adjacency matrix. The percentage of each node types in each region are shown as in **Fig. 3**. The results are similar to those for preprocessing 1 and a threshold of Z=2.56 (**Fig. 5**), in that there was a higher percentage of global hubs in the frontal regions. *p<0.05; **p<0.01; ***p<0.001 by permutation test. Chi-square test results are shown by p values (corrected with Bonferroni method) in each graph.

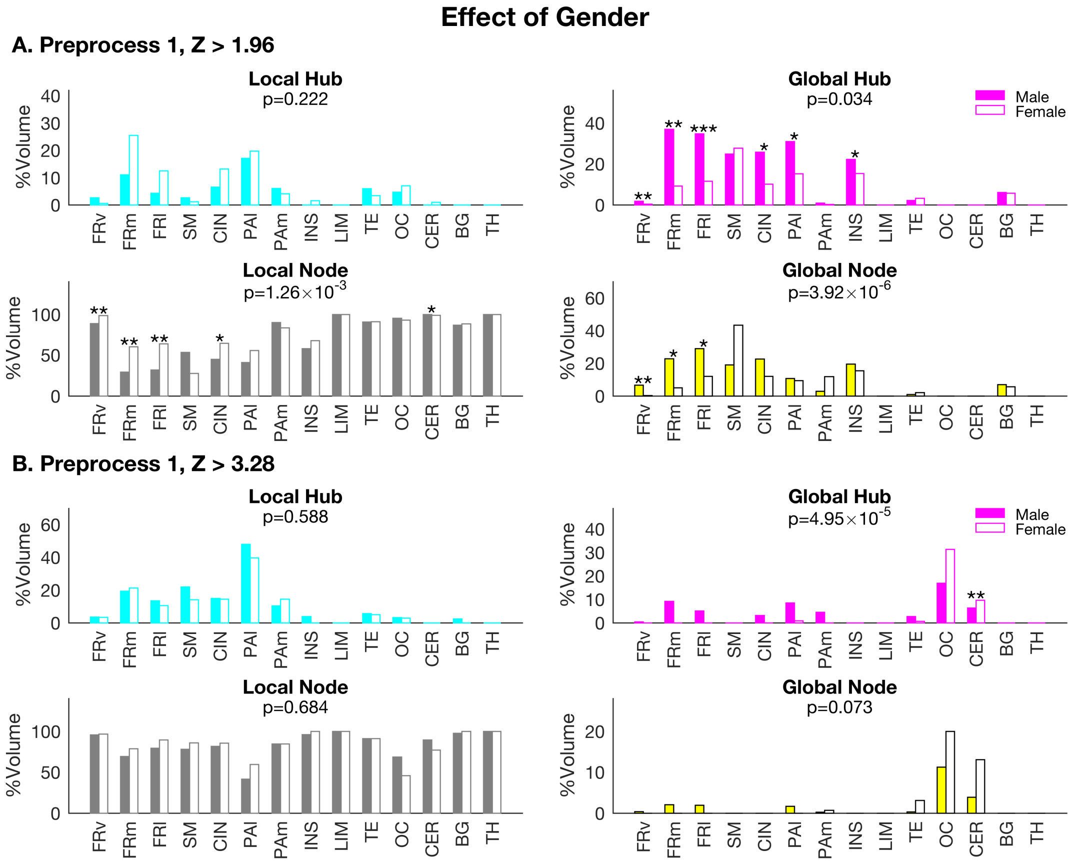

Figure S7. The effect of preprocessing upon network variation with gender. Gender difference in the node type distributions were investigated using the data from preprocessing 2 with a threshold of Z = 2.58 for an adjacency matrix. The percentage of each node types in each region are shown as in **Fig. 3**. The results were similar to those for preprocessing 1 and for a threshold of Z = 2.56 (**Fig. 5**) in that there was a higher percentage of global hubs in the frontal regions. *p<0.05; **p<0.01; ***p<0.001 by permutation test. Chi-square test results are shown by p values (corrected with Bonferroni method) in each graph.

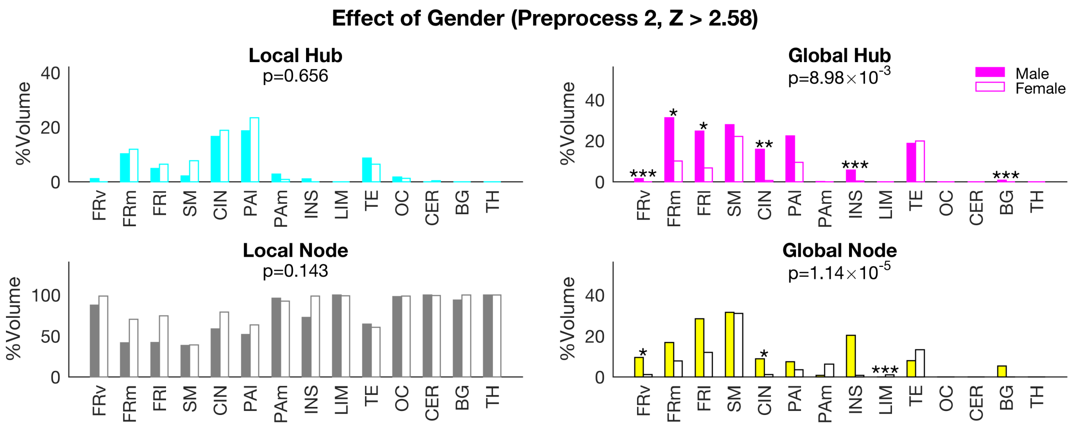

Figure S8. The effect of preprocessing upon network variation with digit ratio. The effects of digit ratio were investigated using the data for males (top) and females (bottom) with preprocessing 2 and a threshold of Z = 2.56 for an adjacency matrix. The percentage of each node types in each region are shown as in **Fig. 3**. *p<0.05 by permutation test. Low: low digit ratio group; High: high digit ratio group. Chi-square test results are shown by p values (corrected with Bonferroni method) in each graph.

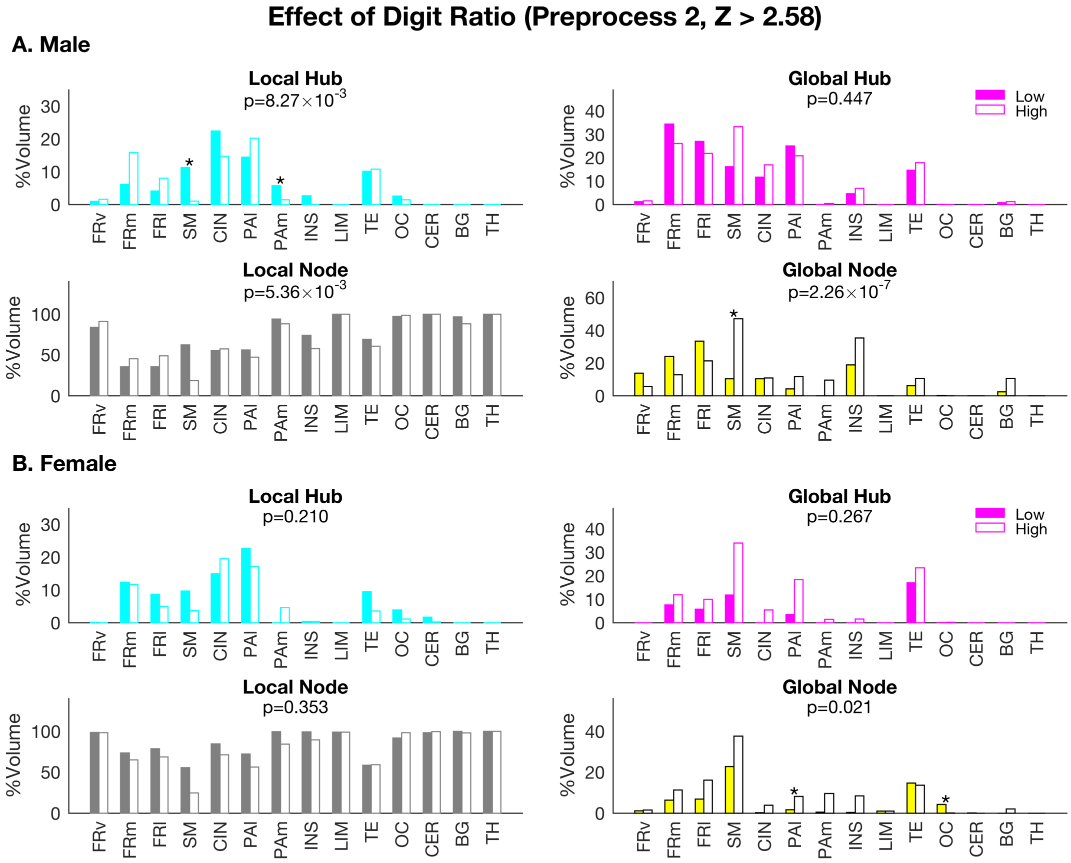

Figure S9. The effect of preprocessing upon network variation with menstrual phase.

The effect of menstrual phase on the network property distributions are investigated using the data for low and high digit ratio groups with preprocessing 2 and a threshold of Z = 2.58 for an adjacency matrix. The percentage of each node types in each region are shown as in **Fig. 3**. *p<0.05; ***p<0.001 by permutation test. Chi-square test results are shown by p values (corrected with Bonferroni method) in each graph. FOL: follicular phase; LUT: luteal phase.

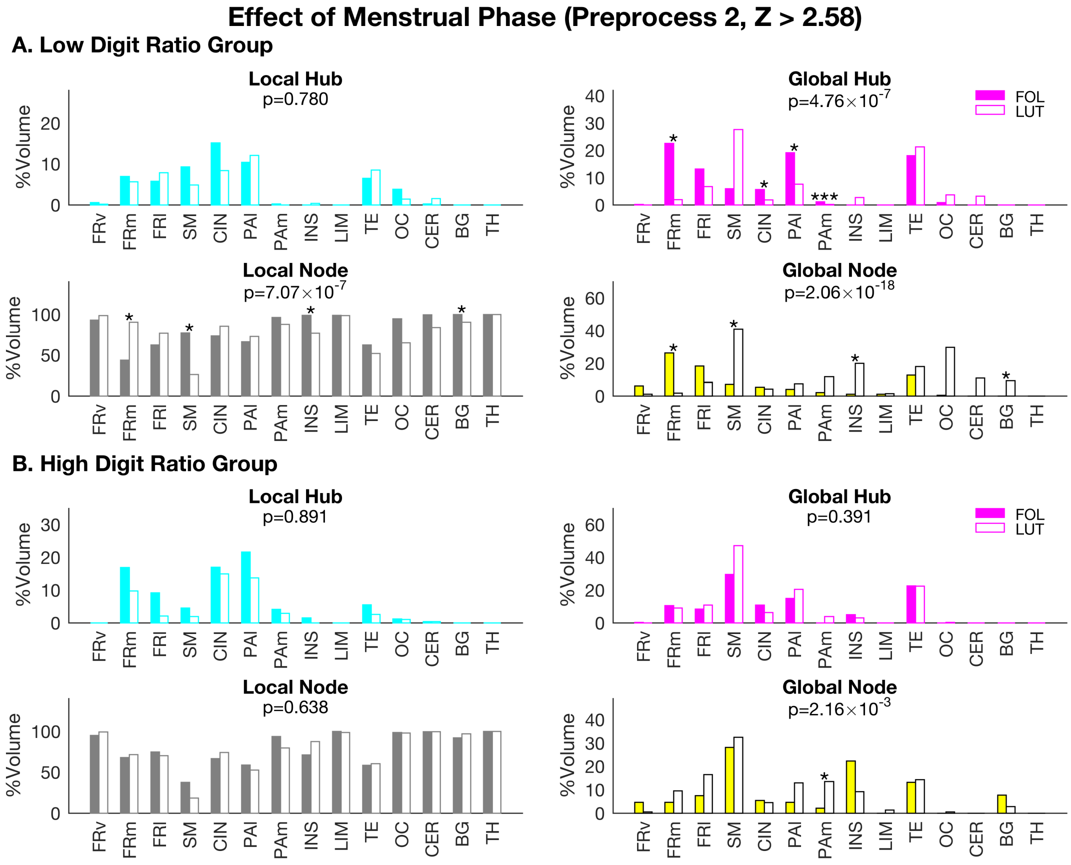

References

Bright, M.G., Murphy, K. (2015) Is fMRI "noise" really noise? Resting state nuisance regressors remove variance with network structure. NeuroImage, 114:158-69.

Liu, T.T. (2016) Noise contributions to the fMRI signal: An overview. NeuroImage, 143:141-151.

Liu, T.T., Nalci, A., Falahpour, M. (2017) The global signal in fMRI: Nuisance or Information? NeuroImage, 150:213-229.

Power, J.D., Plitt, M., Laumann, T.O., Martin, A. (2016) Sources and implications of whole-brain fMRI signals in humans. NeuroImage.

Pujol, J., Macia, D., Blanco-Hinojo, L., Martinez-Vilavella, G., Sunyer, J., de la Torre, R., Caixas, A., Martin-Santos, R., Deus, J., Harrison, B.J. (2014) Does motion-related brain functional connectivity reflect both artifacts and genuine neural activity? NeuroImage, 101:87-95.
